# Amputation of Lower Jaw, with Disarticulation of Both Condyles—On the Claims of Priority in the Exsection and Disarticulation of the Lower Jaw—An Address to the Graduates of the Medical Department of the St. Louis University—Practical Chemistry a Branch of Medical Education—An Essay on Empirical Remedies

**Published:** 1852-08

**Authors:** 


*Amputation of Lower Jaw, with Disarticulation of both Condyles,*—by J. M. CARNOCHAN, M. D.

*On the Claims of Priority in the Exsection and Disarticulation of the Lower Jaw, with an Appendix containing the Report of several Operations performed by* GEO. C. BLACKMAN, M. D.

*An Address to the Graduates of the Medical Department of the St. Louis University's Session* 1851–52,—by CHAS. A. POPE, M. D, Prof. of Surgery.

*Practical Chemistry, a branch of Medical Education, considered in a brief Letter to his Class,*—by ALFRED L. KENNEDY, M. D. Lecturer in the Philadelphia School of Medicine, &c.

*An Essay on Empirical Remedies, read before the Medical Society of the State of Georgia, April,* 1852,—by ROBT. CAMPBELL, M. D., Chairman of the Committee on Empirical Remedies.

Dr. CARNOCHAN, in his pamphlet, claims for himself the credit of being the first to remove successfully the entire lower jaw, and for dupuytren, the honor "of having in 1812 first removed by a methodical operation a portion of the body of the inferior maxilla." Plates, accompanying the report of the case, show the appearance of the bone after maceration, and also the appearance of the patient four months after the operation.

Dr. Blackman, in opposition to Dr. Carnochan, claims for Dr. Deadrick, of Rogersville, Tennessee, the glory of performing the first methodical operation for the removal of a portion of the lower jaw. The operation of Dr. Deadrick was performed in 1810, and reported in the *American Medical Recorder* for July, 1823. The operation to which Prof. Carnochan alludes as accredited to Walther, of Bonn, but which he is unable to trace to an official source, seems to be fully authenticated by Dr. Blackman.

From the address of Prof. Pope we cannot forbear making a short extract. In defending medicine against the charge of uncertainty, he speaks like a man who knows and feels the dignity of his position. After having alluded to the complex nature of the subject of medical enquiry, and to the gradual process by which the human mind masters the unknown, he says:—

"We declare, then, that although to a great extent based on the calculus of probabilities, there is much certainty in medicine, both as a science and an art—that it is a profession founded on correct general principles, and guided by scientific rules of action. There is enough, amply to repay its zealous cultivators, enough to maintain its claims to the respect and confidence of mankind, and even to justify its title of divine. The ancients so thought, in associating it with light, and wisdom and music, and placing them all under the care of Apollo, the god of the sun. Sanctioned, too, by the example and precept of the Saviour of man, its claims to our gratitude and love are surely binding and eternal. Being so accustomed to its aid and benefits, the public are hardly aware of the great good our profession is capable of conferring. But some idea of its proper appreciation may be formed, when we observe the consideration paid to the scientific physician by nations who are uncivilized. By such he is regarded as an almost supernatural being, in whose hands are the issues of life and death. And who is bold enough to deny that such is not often the case? Even among less barbarous people, he is often looked upon as weilding power almost superhuman. See, during the sixteenth century, the French soldiers at the seige of Metz, refusing to fight, until, "our Pare is with us," then, with this, their war-cry, rush to conquer. See Grant, the surgeon missionary, among the benighted nations of the East, armed with his needle for cataract, and winning his way through contending hosts and guarded passes, where armies dare not venture.

"An argument in favor of the alleged uncertainty of medicine, is frequently derived from the differences among physicians themselves, in respect to the nature and treatment of a given malady. It were easy to show the utter fallacy of such an objection, but without entering into a labored proof on this point, I will merely state that what, to the public, may appear discord and opposition, is but apparent only, and to the scientific physician perfectly reconcilable and rational. As in mechanics or mathematics, the same problem will scarcely ever be regarded in precisely the same light, by any two minds, so in medicine, the same end may be reached by different means. There are many ways to Rome."

That medicine is not stationary, is shown by reference to the diseases of the chest. The desponding exclamation of Baglivi, "O quanto difficile morbos pulmonum curare, O quanto difficilius eosdem cognoscere," is no longer true. The greatest difficulty now is, not to *diagnose* but to *cure* diseases of the lungs.

The whole address is full of thought as well as beauty.

The value to the medical man of practical chemistry is demonstrated, we think, by Dr. Kennedy. While he shows a zeal for his own department of our science, he does not claim for it an undue importance.

Dr. Campbell's address on empirical remedies is an able exposition of the devices by which the public mind is duped by nostrum venders. The conclusion arrived at is, that the principal elements of the existence of quackery "consist in—1st, the general ignorance pervading the public on all medical subjects; and, 2d, the imperfect and indiscreet arrangement of the legislative regulations of this country concerning subjects of medical interest." The remedy proposed is, the publishing of the pernicious effects of quackery, and taking the necessary measures to secure the repeal of that portion of the "patent law" referring to secret remedies.

